# Physiological responses of young thoroughbred horses to intermittent high-intensity treadmill training

**DOI:** 10.1186/1751-0147-55-59

**Published:** 2013-08-17

**Authors:** Hajime Ohmura, Akira Matsui, Tetsuro Hada, James H Jones

**Affiliations:** 1 Current address: Sports Science Division, Equine Research Institute, Japan Racing Association, 321-4 Tokami-cho, Utsunomiya-shi, Tochigi 320-0856, Japan; 2 Department of Surgical and Radiological Sciences, School of Veterinary Medicine, University of California, Davis CA 95616, USA; 3 Hidaka Research and Training Center, Japan Racing Association, 535-13 Nishicha, Urakawa-gun, Urakawa-cho, Hokkaido 057-0171, Japan

**Keywords:** *V*o_2_max, Equine, Training, Exercise, Lactate, Heart rate

## Abstract

**Background:**

Training of young Thoroughbred horses must balance development of cardiopulmonary function and aerobic capacity with loading of the musculoskeletal system that can potentially cause structural damage and/or lameness. High-speed equine treadmills are sometimes used to supplement exercise on a track in the training of young Thoroughbreds because the horse can run at high speeds but without the added weight of a rider. We tested the hypothesis that intermittent high-intensity exercise on a treadmill of young Thoroughbred horses entering training can enhance development of aerobic capacity (*V*o_2_max) and running performance more than conventional training under saddle, and do so without causing lameness.

**Results:**

Twelve yearling Thoroughbreds trained for 8 months with conventional riding (C) only, conventional riding plus a short (2 month, S) interval of once-per-week high-intensity treadmill exercise, or a long (8 month, L) interval of once-per-week high-intensity treadmill exercise. Three treadmill exercise tests evaluated *V*o_2_max, oxygen transport and running performance variables in June of the yearling year (only for L), October of the yearling year and April of the 2-year-old year. No horses experienced lameness during the study. Aerobic capacity increased in all groups after training. In both October and April, *V*o_2_max in L was higher than in C, but did not differ between L and S or S and C. Running speeds eliciting *V*o_2_max also increased in all groups after training, with S (809 ± 3 m/s) and L (804 ± 9 m/s) higher than C (764 ± 27 m/s). Maximum heart rate decreased for all groups after training. Hematocrit and hemoglobin concentration increased for L throughout training.

**Conclusions:**

Young Thoroughbred horses can increase aerobic capacity and running performance more than by strictly using track training under saddle with the addition of intermittent high-intensity treadmill exercise, and they can do so without experiencing lameness. This finding suggests that young racehorses might be able to achieve higher aerobic fitness during training without subjecting their musculoskeletal systems to increased loading and risk of developing lameness. The findings of this preliminary study do not indicate a specific protocol to best achieve this goal.

## Background

Training of young Thoroughbred horses must be conducted conservatively to prevent injuring the animals during the period when their musculoskeletal systems are developing and vulnerable. Young Thoroughbreds start to train in the autumn of their 1-year-old year. The first step of training is so-called “breaking” to accustom the horse to being handled and ridden. Following that, training intensity is increased gradually. In the initial stages of training, low-intensity exercise that includes trotting and slow cantering is used to develop aerobic capacity in so-called “endurance training” [1]. In Japan, typical training protocols continue without high-intensity exercise until March of the 2-year-old year. Horse trainers in Japan believe it is essential for preventing injury not to engage in high-intensity exercise during this initial period.

High-speed treadmills for horses are primarily used for clinical evaluation and research. It is easy to control exercise intensity on a treadmill by changing running speed and inclination, and horses running on a treadmill typically do not carry the weight of a rider, so leg loading is reduced. Therefore, it is possible that treadmill training might be utilized as an adjunct training tool to engage young Thoroughbreds in high-intensity exercise to develop aerobic capacity without putting their locomotor systems at higher risk. The purpose of this study was to determine if young Thoroughbred horses trained with intermittent high-intensity treadmill exercise added to a typical track-training routine would develop aerobic capacities different than those trained strictly conventionally under saddle on a track. Furthermore, we evaluated if the addition of such intermittent training could be done without causing lameness in the young horses.

## Methods

Protocols for the study were reviewed and approved by the Animal Welfare and Ethics Committee of the Japan Racing Association (JRA).

### Horses

Twelve Thoroughbreds (seven males, five females, average body weight 400 ± 29 (SD) kg, age 13.6 ± 1.0 month at the start) were studied. The horses underwent a preliminary surgery in April of their yearling year to move a carotid artery from the carotid sheath to a subcutaneous location to facilitate arterial catheterization. After recovery from the surgeries, the horses were trained to run on a motorized treadmill^a^ while wearing an open-flow mask. At least one month passed between the surgery and first treadmill experiments.

### Training groups

Horses were randomly split into three groups for training. All groups were trained identically under saddle on the track. A control group (C) trained only under saddle (conventional training). A short group (S) trained with intermittent (once-per-week) high-intensity exercise on a treadmill for 2 months and a long group (L) also trained with weekly intermittent high-intensity exercise on a treadmill but for 8 months.

### Training on track under saddle

**Figure 1:**
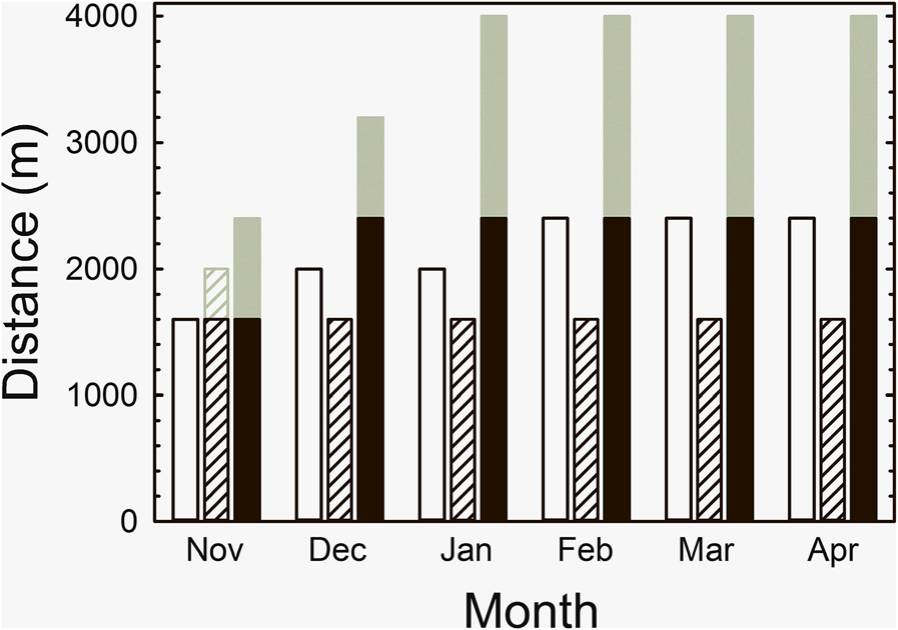
**Schematic of training program under saddle for yearling/2-year-old horses in the study.** For each month, bars show distances horses covered during daily workouts on an oil-sand track at the walk (open bars), trot (hatched bars) and canter (solid bars). Areas in gray indicate variations in distance depending on speed. Cantering was < 10 m/s during Nov and Dec, and then gradually increased to a maximum speed of approx. 13.5 m/s in April for distances up to 1,000 m.

A summary of the training program on the track is in Figure 1. All horses were broken to handling and riding from September to October of their yearling year and then began exercising on an oil-sand track at a trot and slow canter. Running speed was < 10 m/s and total canter distances were 1,600-2,400 m during the yearling year. At the end of the yearling year, canter distances increased to 2,400-4,000 m with 600–1,000 m at speeds of 10 m/s. After February of their 2-year-old year, speeds were gradually increased further to 13.5 m/s for distances of up to 1,000 m.

### Training on high-speed treadmill

The S and L groups received identical riding training on the track as C; however, they also trained once a week with high-intensity exercise by running on a treadmill. Horses were not ridden on days they ran on the treadmill. The S group received treadmill training from February to April of their 2-year-old year, L from August of their yearling year (before breaking) to April of their 2-year-old year. Horses in both groups trained on the treadmill by running up a 6% incline once a week. Running speed on the treadmill was set to elicit exhaustion between 2.5-3.0 min. When a horse could run over 3.0 min, running speed on the treadmill was increased by 0.5 m/s the following week.

### Lameness evaluation

Horses were examined by a minimum of three JRA veterinarians at least once each week (usually more frequently) throughout the study for signs of lameness. Each of the examining veterinarians had a minimum of three years of experience working as a clinician at a JRA training center or racetrack with daily duties including lameness examinations of horses. Horses were evaluated for lameness by observation while being led in hand at walk or trot on a hard surface and while performing their treadmill training.

### Treadmill measurements

Three exercise tests were performed to measure oxygen transport variables for each horse: June of the yearling year (L only), October of the yearling year and April of the 2-year-old year. During these tests, horses ran at steady-state to quantify oxygen transport variables at speeds up to and including the speed that elicited their maximum rate of oxygen consumption (*V*o_2_max). The speed at which the test was performed was intended to be 110-115% of that required to elicit *V*o_2_max in that horse, insuring that O_2_-transport variables would be maximized. For measurements in April for S and L, as well as those in October for L, the speed was determined from the previous weeks’ training run as the speed that the horse could maintain for just over 2 min. For the initial measurement run on each horse, as well as in April for C, the required speed was estimated with an incremental step test that the horses performed the previous week. The protocol consisted of a warm-up (1.8 m/s for 2 min and 3.0 m/s for 3 min) followed by 2-min exercise intervals at 1.8, 4.0, 6.0, 8.0, 10.0, 12.0 and 13.0 m/s, all with the treadmill inclined to a 6% grade. The speed that the horse could maintain for just over 2 min was estimated from those results, as we have previously observed that the highest speed attained with this step-test protocol typically elicits 110-115% *V*o_2_max when horses are run with a protocol in which they are taken to high speed following a warm-up (unpublished data).

Before leading a horse onto the treadmill, an 18 ga × 6.4 cm Teflon catheter^b^ was placed in the horse’s carotid artery and an 8.5 Fr × 9 cm introducer^c^ and 6 Fr × 9 cm introducer^d^ in a jugular vein. A Swan-Ganz catheter^e^ was passed via the jugular introducer so that its tip was positioned in the pulmonary artery, confirmed by measuring pressure at its tip with a pressure transducer^f^.

After catheters and transducers were connected and tested, the horse began its warm-up with 2 min of walking (1.8 m/s) and 3 min of trotting (3.5 m/s). For these experiments, horses exercised with the treadmill inclined to a 6% grade for 2 min each at speeds of 1.8, 4.0 and 7.0 m/s, and then ran at the speed that elicited *V*o_2_max in that horse, in the range of 11.8-13.5 m/s. The exercise protocol was terminated when a horse could no longer maintain its position near the front of the treadmill. During this procedure, the horses wore an open-flow mask for measurement of oxygen consumption (*V*o_2_) and electrocardiogram electrodes for measuring heart rate (*HR*).

As the horse exercised at each speed after coming to steady state for *V*o_2_, arterial (a) and mixed-venous $\left(\bar{v}\right)$ blood samples were drawn simultaneously for measurement of blood hemoglobin concentration ([*Hb*], oxygen saturation (*S*o_2_) and oxygen concentration (*C*o_2_) using a hemoximeter set to its equine algorithm^g^. Samples of blood were centrifuged^h^ (12,000 × *g*) to measure packed cell volume (*PCV*). In conjunction with *V*o_2_ measured by the open-flow system, cardiac output (*Q*) was calculated using the Fick Principle and cardiac stroke volume (*V*_S_) as the quotient of *Q* and *HR*. Plasma lactate concentration ([*LA*]) was measured with a lactate analyzer^i^. Heart rate averaged during the last 10 s at each speed was determined from electrocardiogram recordings made using bipolar electrodes across the long axis of the heart that were amplified^j^ and recorded on a personal computer using A/D hardware^k^ and software^l^ sampling at 500 Hz.

### Oxygen consumption

Horses wore a 25-cm diameter open-flow mask on the treadmill through which a rheostat-controlled 3.8-kW blower drew air at bias flow rates of 6,000–8,000 l (ATP)/min. Air flowed through 20-cm dia wire-reinforced flexible tubing affixed to the mask and across a 25-cm dia pneumotachograph^m^ connected to a differential pressure transducer^n^; this was used to ensure that the bias flows during measurements were identical to those during calibrations. Oxygen consumption was measured with standard mass-balance techniques [2,3] using an oxygen and carbon dioxide analyzer^o^, 2-m long Nafion drying tube with countercurrent dry gas flow (Drierite (CaSO_4_)) to remove water from sample gas, and mass flowmeters^p^ for measuring nitrogen and carbon dioxide calibration flows using the N_2_-dilution/CO_2_-addition technique [4]. Gas analyzer and flowmeter outputs were also recorded with A/D hardware^k^ and software^l^ on personal computers.

### Measurement of running velocity eliciting heart rate of 200 beat/min (*V*_200_)

The running velocity that elicited a heart rate of 200 beat/min (*V*_200_) [5] was estimated at the end of training in April of the horses’ 2-year-old year by running them on an 800-m circular indoor oil-sand track. Heart rate during exercise was recorded with a *HR* meter^q^. Running speeds were calculated by dividing a measured distance travelled (200 m) by the elapsed time. After warming up for 800 m at a trot, horses ran for 800 m at four different speeds: approx. 3.4, 7.7, 9.2 and 11.2 m/s. The *V*_200_ was extrapolated from a regression of *HR* and running speed. If *HR* exceeded 210 beat/min, that datum was excluded to preclude the possibility of biasing the data by including a maximum *HR*.

### Statistical analysis

Results are expressed as mean ± SD. Two-way repeated measures ANOVA tested for differences between treatment groups at different times using group and time as factors. One-way repeated measures ANOVA tested for differences with time in the L group because it included an extra sampling period during the first year. The Holm-Šidák procedure was used for *post-hoc* pairwise comparisons (*P* ≤ 0.05). One-way ANOVA evaluated differences between groups for *V*_200_. Data were analyzed with commercial statistical software^r^.

## Results

### Lameness

No horses in any of the groups experienced lameness during the course of the study.

### Body mass

The body masses (*M*_b_) of horses in the three groups averaged within 5% of each other over the entire study and did not differ significantly between groups at any time period (Table 1). Horses gained an average of 66.5 kg (16.6%) during the first interval and lost 16.8 kg (3.6%) during the second, although only the C group lost significant weight during the October-April interval (*P* = 0.015).

**Table 1:** Cardiopulmonary and running performance variables measured in 12 Thoroughbreds during and at the end of a period of initial training

	**June**			**October**			**April**		
	**Control**	**Short**	**Long**	**Control**	**Short**	**Long**	**Control**	**Short**	**Long**
Body mass (*M*_b_) (kg)	417 ± 19^ab^	401 ± 39^cd^	384 ± 24^ef^	490 ± 8^a^	456 ± 25^c^	456 ± 22^e^	461 ± 32^b^	445 ± 35^d^	444 ± 14^f^
Max running speed (m/s)			12.4 ± 0.3^a^	12.3 ± 0.3^b^	12.1 ± 0.2^c^	12.4 ± 0.2^d^	12.7 ± 0.5^b^	13.5 ± 0.1*^c^	13.4 ± 0.2*^ad^
*V*o_2_max / *M*_b_ (ml O_2_ (STPD)/(min kg)			133 ± 3^a^	127 ± 5^b^	131 ± 2^c^	149 ± 12*^d^	148 ± 9^b^	158 ± 10^c^	172 ± 16*^ad^
*HR* (beat/min)			243 ± 4	231 ± 3	239 ± 4^c^	237 ± 11	222 ± 2	224 ± 5^c^	228 ± 3
_*Q* (1/min)_			260 ± 31	305 ± 5	310 ± 35	315 ± 32	326 ± 27	328 ± 22	329 ± 43
*Q*/*M*_b_(ml/min kg)			676 ± 44	622 ± 14^a^	680 ± 40	690 ± 53	707 ± 40^a^	739 ± 41	739 ± 73
*V*_S_ (l)			1.07 ± 0.14^a^	1.32 ± 0.04^b^	1.30 ± 0.15^c^	1.33 ± 0.11	1.47 ± 0.11^b^	1.47 ± 0.10^c^	1.44 ± 0.19^a^
*V*_S_/*M*_b_ (ml/kg)			2.79 ± 0.21	2.70 ± 0.09^a^	2.84 ± 0.19^b^	2.92 ± 0.20^c^	3.18 ± 0.16^a^	3.30 ± 0.17^b^	3.24 ± 0.32^c^
[*Hb*] g/dl			18.2 ± 1.4^a^	18.8 ± 0.6	18.7 ± 0.4^b^	19.5 ± 1.6 ^c^	19.4 ± 0.8	19.6 ± 0.1^b^	21.6 ± 0.8*†^ac^
*PCV* (%)			48.3 ± 3.1^a^	52.0 ± 0.8	53.5 ± 2.6	52.3 ± 3.0^b^	53.0 ± 1.2	54.6 ± 0.5	57.5 ± 2.6*†^ab^
*C*_a_O_2_ (ml O_2_ (STPD)/d			21.6 ± 0.6^a^	22.8 ± 1.1	23.3 ± 0.7	23.0 ± 1.0^b^	22.8 ± 2.1	23.6 ± 0.8	25.6 ± 1.8^ab^
$C_{\bar{v}}O_{2}\;\left(\mathrm{ml}\;O_{2}\;\left(\mathrm{STPD}\right)/\mathrm{dl}\right)$			1.9 ± 0.7	2.4 ± 0.1	2.8 ± 0.3	1.4 ± 0.5*†	2.4 ± 0.5	2.3 ± 0.4	2.0 ± 0.4 *
*S*_a_O_2_ (%)			88.0 ± 4.3	87.1 ± 4.3	91.9 ± 2.1	86.7 ± 5.1	91.0 ± 5.4	89.35 ± 3.1	85.7 ± 2.6
$S_{\bar{v}}O_{2}\;\left(\%\right)$			8.1 ± 3.6	9.3 ± 2.8	11.2 ± 1.4	6.8 ± 3.3	10.1 ± 1.8	8.8 ± 1.8	6.8 ± 1.2
[*LA*] (mmol/l)			12.6 ± 1.3	18.3 ± 4.8	15.0 ± 1.7	14.6 ± 5.3	15.8 ± 2.6	16.2 ± 2.9	17.2 ± 4.1
*V*_200_ (m/min)							613 ± 66	652 ± 70	696 ± 30

Measurements were made running on a treadmill at a 6% incline while running at maximal aerobic capacity. Exercise tests were conducted in June and October of the yearling year and April of the 2-yr-old year. Experimental treatment groups were Control (conventional training under saddle on track only), Short (conventional training under saddle on track with one day per week of high-intensity treadmill exercise for 2 mo) or Long (conventional training under saddle on track with one day per week of high-intensity treadmill exercise for 8 mo). Variables measured or calculated were body mass (*M*_b_), speed to elicit maximum O_2_ consumption (*V*o_2_max), *V*o_2_max/*M*_b_, heart rate (*HR*), cardiac output (*Q*), *Q*/*M*_b_, cardiac stroke volume (*V*_S_), *V*_S_/*M*_b_, hemoglobin concentration ([*Hb*]), hematocrit (*PCV*), arterial O_2_ concentration (*C*_a_O_2_), mixed-venous O_2_ concentration $\left(C_{\bar{v}}O_{2}\right)$, arterial O_2_ saturation (*S*_a_O_2_), mixed-venous O_2_ saturation $S_{\bar{v}}O_{2}$, end-run plasma lactate concentration ([*LA*]) and running speed on track with rider required to elicit heart rate of 200 beat/min (*V*_200_). * Indicates values are significantly different from the C group. ^†^ Indicates values are significantly different from the S group. Identical superscripts (^a, b, c, d, e, f^) indicate values are significantly different from other values with the same superscript (*P* ≤ 0.05).

### Running speed and maximum O_2_ consumption

Maximum running speeds increased in all groups after training with significant interaction (*P* = 0.003) between group and time. In the final test in April, both treadmill exercise groups (S: 13.5 ± 0.1 m/s, *P* = 0.002; L: 13.4 ± 0.1 m/s, *P* = 0.003) were higher than C (12.9 ± 0.4 m/s). The specific *V*o_2_max of all groups increased between measurement periods (*P* < 0.001) and the groups differed from each other (*P* = 0.014). The L horses were more highly aerobic than C (*P* = 0.015) at both times, and verged on being significantly greater than S (*P* = 0.065). The S and C groups did not differ (*P* = 0.280) (Table 1).

### Heart rate, cardiac output and stroke volume

Maximum *HR* decreased by an average of 10.8 beat/min (4.6%) between October-April (*P* = 0.002) in all groups with no differences between groups (Table 1). Whole-body *Q* did not differ between groups nor over time, but *Q*/*M*_b_ increased by an average of 64.2 l/(min kg) (9.7%) between the final two tests (*P* = 0.006). Cardiac *V*_S_ increased by an average of 0.143 l (10.9%) between tests (*P* = 0.001), as did *V*_S_/*M*_b_ (0.321 ml/kg, 14.9%, *P* < 0.001).

### Hemoglobin concentration and hematocrit

The [*Hb*] at *V*o_2_max showed significant interaction between group and time, increasing by an average of 1.19 g/dl (6.3%) for all groups between October-April. There was no difference between groups in October; however, the L group was higher than C and S by April. The L group increased [*Hb*] by a total of 3.40 g/dl (18.7%) over its 8 months of training. These changes were confirmed by *PCV* also showing significant interaction between group and time, increasing between October-April by an average of 2.46% (4.7% increase), and for the L group increasing a total of 9.25% for a 19.2% change (Table 1).

### Arterial O_2_ concentration and saturation

The *C*_a_O_2_ showed significant interaction between group and time, with the L group increasing by a total of 3.73 ml O_2_ (STPD)/dl (17.2%) over the entire period of training (*P* = 0.001) and increasing during both intervals (*P* = 0.050 and *P* = 0.010, respectively). The *S*_a_O_2_ did not differ between time periods (*P* = 0.898) nor between groups (*P* = 0.194).

### Mixed-venous O_2_ concentration and saturation

The $C_{\bar{v}}O_{2}$ showed significant interaction between group and time, with the L group averaging approx. 0.9 ml O_2_ (STPD)/dl less than C and S, although pairwise comparisons were only significant in October (*P* = 0.038 and *P* = 0.007, respectively). The $S_{\bar{v}}O_{2}$ differed between treatments (*P* = 0.050), but pairwise comparisons only indicated that the L group verged on being significantly lower than C and S (*P* = 0.078 and *P* = 0.079, respectively).

### End-run lactate concentration and velocity eliciting heart rate 200

End-run plasma lactate concentration did not differ between groups (*P* = 0.824) nor times (*P* = 0.655). The *V*_200_ also did not differ between groups (*P* = 0.179), although the test had very low power to detect differences (power = 0.181).

## Discussion

Most of the energy expended over the distances and durations of Thoroughbred flat races is generated aerobically [6]. Therefore, developing aerobic capacity is a fundamental goal of training for young Thoroughbreds. We undertook this study to determine if young horses beginning race training that received intermittent high-intensity treadmill exercise could enhance their aerobic capacities without developing lameness problems from potential associated increased stresses on that system. We hypothesized that a combination of conventional training under saddle and intermittent high-intensity training on an inclined treadmill would produce sufficient stimulus to enhance development of aerobic capacity and running performance of young Thoroughbreds without increasing limb loading sufficiently to damage their musculoskeletal structures and induce lameness. It is well known that relatively few repetitions of limb loading at modest intervals of time are sufficient stimulus to cause bone to remodel to support the load, so it doesn’t have to be frequently stressed heavily to strengthen and maintain its remodeling [7].

### Comparisons with other studies

It is inherently difficult to compare results of different training studies because different studies use horses of different ages, training histories, and training intensities and durations [1,8-21]. All of these factors are likely to influence the physiological responses of the animals to the exercise program. For that reason, we focus our discussion of comparisons between training responses of the horses in this study to those of horses of similar age that were trained with similar methods to those of C in this study [20,21]. The temporal comparisons in that study between trained and untrained horses were for training periods of 6 months (October-May, T1-T2) and 12 months (October-October, T1-T3), so the periods differ slightly in timing and duration.

### Sample sizes

The groups used in this study were small, resulting in lower than desired power for many of the statistical tests. Caution should be used when interpreting a lack of significant difference for a number of tests, particularly those that verged on being significant even with the small sample size used and associated low power. Nevertheless, a number of significant differences were detected between groups or time periods. Further evaluation of hypothesis tests related to mechanisms responsible for the effects observed in these studies should be done with larger groups.

### Energy expenditure for different protocols

We estimated the difference in workload that horses experienced with incremental treadmill exercise *vs.* their track workouts by approximating their costs of transport based on speed, incline and weight carried and multiplying those by the distances/times they ran at them. Taylor [22] demonstrated that energy cost of transport (J/(m kg)) is relatively independent of running speed. Energy cost is directly proportional to weight carried [23] and the equations of Schroter and Marlin [24] can be used to estimate energy expenditure due to incline. Given that the horses in this study had similar body masses between the last two tests and that their fastest track and treadmill speeds were both approx. 13.5 m/s, we can estimate that carrying approx. 60 kg of rider and tack during track training would increase energy cost for a 450 kg horse by approx. 13.3% at a given speed, whereas, running up a 6% incline unloaded at that same speed would increase metabolic power required by approx. 76.1%. This difference in energy expenditure is presumably what stimulated the differences observed in aerobic capacity between groups. However, the total sum of energy required to move the horses over their 8,000 m track workout for group C at the end of training (Figure 1) *vs.* the 4,812 m treadmill protocol for L and S was approx. 6.97% higher (approx. 10.1 MJ *vs.* 9.4 MJ). Rate of energy expenditure appeared to be a more important stimulus for the observed results in aerobic capacity than the total energy expended.

### Lameness

None of the horses that were exercised with intermittent high-intensity treadmill running showed signs of lameness during the study. Given that JRA horses of this age undergoing conventional training typically experience a low incidence of lameness (< 5%), it would require a considerably larger sample size to determine if treadmill-trained horses truly do not differ in their incidence of lameness than conventionally-trained horses. However, for the purposes of answering the fundamental question asked in this preliminary study, it is notable that intermittent high-intensity treadmill exercise induced lameness in none of these horses, even in those that experienced it for 8 months.

### Body mass

Horses grew during the June-October period, as expected for their age, and only the C group changed in body mass during the October-April training period. These results are similar to those reported for other yearlings trained similarly [20].

### Running speed and maximum oxygen consumption

Training is designed to increase sustainable running speed as aerobic capacity increases [1]. Both of these outcomes occurred in these horses, although not uniformly for different treatment groups. Both groups that received intermittent high-intensity treadmill training reached *V*o_2_max at higher speeds (> 0.7 m/s) than the C group by the final exercise test. The speeds required for the C group horses to reach *V*o_2_max in this study in April were close to those observed previously in horses that had trained by similar protocol for one month longer (12.7 *vs.* 12.9 m/s, respectively) [20]. Furthermore, although *V*o_2_max of the horses increased in all groups after training, as expected, the L group increased significantly more than C, and verged on doing so compared to S (Table 1). It is curious that running speed to achieve *V*o_2_max was essentially identical for the S and L groups, yet *V*o_2_max was 8.86% higher for the L group and verged on being significantly higher (*P* = 0.065). Following one extra month (17–20% additional) of conventional training, horses in a previous study had similar *V*o_2_max/*M*_b_ as the C group in this study in April (156 *vs.* 148 ml O_2_ (STPD)/(min kg), respectively) [20]. It is clear from these results that the additional stimulus of high-intensity training in the L group significantly increased *V*o_2_max and running speed to achieve it, and it appears horses in the S group shared at least some of the performance benefits associated with treadmill training.

### Circulatory variables

The decrease in *HR* seen for all groups between tests in this study may have been related to the decrease in *HR* reported with ageing for horses in general [25], although the 4.6% decrease in these horses during just the October-April period was greater than the 3.7% decrease reported from October–October in a previous study of horses of similar age [20]. The *V*_S_ and *V*_S_/*M*_b_ increased in both the C group (11.4% and 17.8%) and S group (13.1% and 16.2%) between October-April and was 11.0% higher for *V*_S_/*M*_b_ for the L group. When evaluated over the entire period of training, *V*_S_ increased in the L group from June-April. A previous study of horses trained with a protocol similar to that of the C group found significant increases in *Q*/*M*_b_ and *V*_S_/*M*_b_ during the initial 6 months of training, with increases of 13.3% and 14.7%, respectively. These findings suggest that a component of the increased delivery of O_2_ that raised *V*o_2_max with training was increased convective delivery of blood and oxygen.

### Blood O_2_ capacitance, concentration and saturation

The increases in [*Hb*] seen in the S and L groups in this study did not occur in horses trained similarly under saddle and reported previously [20]. Horses in that study did increase *PCV* during their final 6 months of training, similar to the L group horses in this study; however, the [*Hb*] response to training appears to be somewhat variable and may depend on factors, *e.g.*, age and intensity of training. Nevertheless, the 18.7% increase in [*Hb*] and 19.0% increase in *PCV* observed in the L group horses matched the 18.5% increase in *C*_a_O_2_ those horses experienced and likely contributed substantially to the observed 29.3% increase in *V*o_2_max/*M*_b_ these horses achieved. Curiously, $C_{\bar{v}}O_{2}$ was lower in the L group than in C (and S in October). Studies have demonstrated in humans [26], rats [27] and goats [28], and have suggested for horses also [29,30], that peripheral tissue diffusion limitation at *V*o_2_max requires that mean muscle capillary pressure head for diffusion, and hence, $P_{\bar{v}}O_{2}$ and $S_{\bar{v}}O_{2},$ must be higher to increase O_2_ flux for a given muscle diffusing capacity for O_2_. The existence of lower $C_{\bar{v}}O_{2}$ would enhance convective extraction of O_2_, but does not explain how the horses could overcome diffusion limitation unless the L group horses had developed greater muscle diffusing capacity for O_2_, by mechanisms, *e.g.*, increased capillary density. The $S_{\bar{v}}O_{2}$ was lower in the L group than in any other group at all time periods, although those differences were not significant.

### Plasma lactate concentration and *V*_200_

End-run plasma lactate concentrations were similar and did not vary systematically between groups, indicating horses were deriving similar amounts of net anaerobic energy during their runs. Although *V*_200_ averaged progressively lower from the C group to the S group to the L group, those differences were not significant, so this index of aerobic performance did not reflect the observed differences between groups in *V*o_2_max.

## Conclusion

Young horses trained with a conventional track exercise program supplemented with weekly high-intensity treadmill runs that increased their demand for metabolic power by 32% increased aerobic capacity significantly more than those trained only on the track. None of the treadmill-trained horses developed lameness. The results do not indicate if the effectiveness of such a program would vary with age or extent of previous training in young horses or their aerobic capacities at the outset of the training.

## Endnotes

^a^Mustang 2200, Graber AG, Fahrwangen, Switzerland.

^b^Surflow, Terumo, Tokyo, Japan.

^c^MO95H-8.5, Baxter, Tokyo, Japan.

^d^MO95H-6.0, Baxter, Tokyo, Japan.

^e^Criticath, Ohmeda, Madison, WI, USA.

^f^Statham P23d, Viggo-Spectramed, Tokyo, Japan

^g^OSM3 Hemoximeter, Radiometer-Copenhagen, Brønshøj, Denmark.

^h^KH120A, Kubota, Tokyo, Japan.

^i^YSI 2300 STAT Plus, Yellow Springs Instruments, Yellow Springs, OH, USA.

^j^SM-29, Fukuda Denshi, Tokyo, Japan.

^k^DI-720-USB, DATAQ Instruments, Akron, OH, USA.

^l^Windaq Pro+, DATAQ Instruments, Akron, OH, USA.

^m^LF-150B, G. N. Sensor, Chiba, Japan.

^n^TF-105, G. N. Sensor, Chiba, Japan.

^o^METS-900, VISE Medical, Chiba, Japan.

^p^Model DPM3, Kofloc, Tokyo, Japan.

^q^Accurex Plus, Polar Electro Oy, Kempele, Finland.

^r^SigmaPlot 11, Systat, Chicago, IL, USA.

## Abbreviations

Mb: Body mass; Vo2max: Maximum rate of O_2_ consumption; HR: Heart rate; Q: Cardiac output; VS: Cardiac stroke volume; [Hb]: Blood hemoglobin concentration; PCV: Packed cell volume (hematocrit); CaO2: Arterial O_2_ concentration; $C_{\bar{v}}O_{2}$: Mixed-venous O_2_ concentration; SaO2: Arterial O_2_ saturation; $S_{\bar{v}}O_{2}$: Mixed-venous O_2_ saturation; [LA]: End-run plasma lactate concentration; V200: Running speed eliciting a heart rate of 200 beat/min.

## Competing interests

The authors declare that they have no competing interests.

## Authors’ contributions

HO generated the hypothesis and planned the experiment, assisted in data collection, analysis and interpretation, wrote early draft of manuscript. AM and TH assisted with data collection. JHJ assisted in data analysis and interpretation, wrote final draft of manuscript. All authors read and approved the final version of the manuscript.
